# The Rocky Road to viral hepatitis elimination: assuring access to antiviral therapy for ALL coinfected patients from low‐ to high‐income settings

**DOI:** 10.1002/jia2.25073

**Published:** 2018-04-10

**Authors:** Karine Lacombe, Marina B Klein

**Affiliations:** ^1^ Sorbonne Université, INSERM Institut Pierre Louis d’Épidémiologie et de Santé Publique Paris France; ^2^ AP‐HP, Hôpital Saint Antoine Infectious Diseases Unit Paris France; ^3^ Research Institute of the McGill University Health Centre Montréal Québec Canada; ^4^ Division of Infectious Diseases Department of Medicine McGill University Health Centre Montréal Québec Canada

**Keywords:** antivirals, diagnostic, guidelines, hepatitis B, hepatitis C, HIV, key populations, prevention

## 1 Introduction

Chronic viral hepatitis is a leading cause of morbidity and mortality from liver disease worldwide, ranking among the top 10 causes of global mortality in 2013 1. Chronic hepatitis B (CHB) and C (CHC) are responsible for most of this liver disease burden with CHC being predominant in Europe and the Americas and CHB more frequent in the other parts of the world. For years, the response to chronic viral hepatitis has been hampered by lack of public knowledge, inadequate screening policies, poor treatment access and low treatment efficacy 2. However, global inertia has come to a halt in large part due to the advent of curative anti‐HCV direct acting antivirals (DAAs) that have the potential to radically impact the HCV epidemic. The resulting paradigm shift in CHC care and management has mobilized involvement of international global health organizations such as the World Health Organization (WHO) in the fight against viral hepatitis. In 2016, the World Health Assembly of the United Nations called for the elimination of viral hepatitis as a public health threat, with a 90% reduction in cases of viral hepatitis and 65% reduction in mortality by 2030 3. Because of shared routes of transmission, more than four million individuals are estimated to be dually infected with the human immunodeficiency virus (HIV) and either CHC or CHB worldwide 4, 5. Most of these people belong to key populations such as men who have sex with men (MSM) and people who inject drugs (PWID) where specific interventions will need to be implemented to reach elimination of viral hepatitis, including prevention of reinfection. Against this backdrop, a workshop preceding the opening of the ninth IAS conference of HIV Science was held in Paris in July 2017. The meeting brought together researchers studying the epidemiology and modelling of viral hepatitis, professionals caring for those infected, as well as community researchers and advocates. The aim of the meeting was to pave the Rocky Road to viral hepatitis elimination by leaving no one behind (https://www.iasociety.org/Co-Infections/Hepatitis). This special issue of the *Journal of the International AIDS Society* has gathered landmark papers on viewpoints, reviews and original data that were presented and debated during the Workshop. It provides a comprehensive overview of the challenges faced by scientists, stakeholders and the community in addressing questions of why, how and when viral hepatitis will be eliminated, with a specific focus on HIV coinfection and key populations.

As with HIV infection, the approach to viral hepatitis elimination necessitates increasing prevention and implementing action along all the points of the care and treatment cascade. Specific service targets will need to be met along all these points to ensure success. In the commentary by Hutin and colleagues, it is evident that considerable progress is being made with respect to implementing some existing tools for prevention such as improved blood and medical injection safety and birth dose vaccination for HBV, whereas harm reduction targets are falling far short of requirements 6. Diagnosis rates remain appallingly low and without rapid increases in the number of people tested and diagnosed, little progress in the very low treatment rates can be expected.

One of the key aspects for successful implementation of the WHO global health sector strategy on HBV and HCV will be the establishment of national and local policies and programmes that support elimination efforts across sectors. Lazarus and colleagues report on country‐specific responses to viral hepatitis (including public awareness and engagement and the presence of explicit policies for prevention, diagnosis, monitoring and treatment) from the unique perspective of patient groups in Europe (Hep‐CORE) comparing 2016 and 2017 7. While, in general, there was a reported increase in policies and programmes for viral hepatitis over time, more than half of countries did not have national strategies in place to address these epidemics and programming gaps for prevention (e.g. needle exchange) and treatment were notable. The study also highlights the important gaps that remain for engaging civil society in the efforts to eliminate viral hepatitis.

To reach elimination of viral hepatitis, the first major obstacle is identification of the estimated 80% of HCV‐infected persons globally who have not yet been diagnosed. Fourati and colleagues review diagnostic algorithms that might simplify and enhance decentralized diagnostic testing, particularly in low‐ and middle‐income settings 8. For example, the development of reliable HCV core antigen tests and new nucleic acid amplification technologies could permit a one‐step screening and diagnosis strategy. The availability of pangenotypic antiviral therapy may soon obviate the need to perform HCV genotyping prior to treatment which could further simplify management. While promising, these new technologies are currently too costly for widespread deployment.

Once having performed HCV screening in targeted populations, the next critical step is linking those found to be infected to care, retaining them in the healthcare system and ensuring access to treatment. This is particularly challenging in key populations who are at high risk of becoming reinfected (e.g. people who use drugs, men having sex with men) or developing CHC‐associated complications (e.g. HIV coinfected). Sacks‐Davis and colleagues have gathered data from numerous local and national initiatives working towards HCV elimination in HIV coinfected populations globally 9. They show that while treatment has increased substantially in the era of DAAs (mostly in high‐income countries) two‐thirds of people still have not accessed treatment. Even in settings where treatment is largely available, such as most parts of Western Europe, criminalization, discrimination and stigmatization are strong barriers to treatment for all.

One of the major barriers to increasing treatment for HCV has been the high cost of DAAs creating a fundamental paradox: the most expensive antivirals (on a per pill basis) are needed by some of the most marginalized groups least able to advocate for their health. The expansion of HIV therapy has served as a catalyst for change in the financing of anti‐infective therapies and for drug pricing more widely. While the HIV response necessitated the development of creative pricing strategies for brand name drugs and expanded access to low‐cost generic therapies, it is the exorbitant cost of HCV treatment that is forcing a re‐examination of government's role in negotiating prices and the roles of generic companies and NGOs in drug development. Grillon and colleagues propose several practical actions that have been successfully used by treatment advocates that could help increase access to DAAs, especially for people who inject drugs 10.

Alongside the cascade of care for CHC, DAAs are a cornerstone on the road to HCV elimination. The scale up of HCV treatment will require moving treatment beyond specialty settings and necessitate the greater involvement of a broad range of health professionals. In a very practical paper, Aghemo and colleagues review recent treatment recommendations for HCV and provide guidance for clinicians on key management issues including the pretreatment assessment of liver severity, on‐treatment monitoring and follow‐up after reaching sustained virological response 11.

Bringing several lines of evidence together, Martin and colleagues review modelling and cost data for the feasibility achieving HCV elimination in HIV‐positive previously mentioned MSM and previously mentioned PWID 12. They also present a real world example from the Netherlands which demonstrates that a very rapid decline in HCV prevalence can be achieved in HIV‐positive MSM through DAA scale up. Incidence appears to be declining as a result but likely will not reach the 90% reduction in incidence required for elimination. They conclude that elimination is achievable in these key populations, but that treatment alone, despite being cost effective, will be insufficient and must be paired with harm reduction and behavioural changes to prevent reinfections.

Finally, in a commentary that aims to pave the future of research in the field of viral hepatitis, Boyd and colleagues highlight the main evidence gaps that still need to be filled so the United Nations for the Millennium goals of combating viral hepatitis may be reached 13. More tools are needed for preventing ongoing transmission, identifying undiagnosed infections (raising awareness and developing innovative screening tools), to broaden indications for treatment and facilitate access to drugs worldwide, as well as continued investment in the design of new drugs and approaches for HBV cure; all are complementary steps that may eventually lead to hepatitis elimination.

## 2 Conclusion

The availability of safe, all‐oral and curative therapies for HCV is having a transformative influence on the course of the global response to CHC. Lessons learned in striving towards viral hepatitis elimination can also inform responses to HIV, HBV and other emerging infectious disease threats. The expanding response to viral hepatitis has, on the one hand, uncovered health inequities globally, between and within countries, and, on the other hand, provided opportunities to develop new community‐based models of integrated health services that could have wide impact beyond HIV and HCV. At its best, the HCV treatment revolution can be used as a tool to draw marginalized peoples into health services that have frequently been hostile to them and adapt them to their realities thereby acting on a wide array of health and social services needs for vulnerable populations. We are well along the road to viral hepatitis elimination. However, to reach the ultimate goal of elimination, continued mobilization of, and advocacy for, the communities affected, increased investment into research and development of diagnostics and new medicines that are affordable and sustained political engagement will be needed.

## Competing interests

KL participates in advisory boards for Gilead Sciences, Abbvie, Janssen and Merck Sharp and Dome. She receives research grants from Gilead Sciences, Merck and Janssen. MK receives research grants for investigator‐initiated trials from Merck and ViiV Healthcare; consulting fees from ViiV Healthcare, Merck and Gilead Sciences.

## Authors’ contributions

KL and MK conceptualized and outlined the paper. They wrote the introduction, summarized the articles and contributed to the conclusions. Both authors approved the final draft.

## Funding

MK receives funding from the Canadian Institutes of Health Research (FDN‐143270), Fonds de recherche Québec‐ Santé, Réseau SIDA/maladies infectieuses (FRQ‐S), and the Canadian Institutes of Health Research‐Canadian HIV Trials Network (CTN‐222).
